# A benefit-finding intervention for family caregivers of persons with Alzheimer disease: study protocol of a randomized controlled trial

**DOI:** 10.1186/1745-6215-13-98

**Published:** 2012-07-02

**Authors:** Sheung-Tak Cheng, Rosanna WL Lau, Emily PM Mak, Natalie SS Ng, Linda CW Lam, Helene H Fung, Julian CL Lai, Timothy Kwok, Diana TF Lee

**Affiliations:** 1 Department of Psychological Studies, Hong Kong Institute of Education, 10 Lo Ping Road, Tai Po, NT, Hong Kong; 2 Department of Psychiatry, Chinese University of Hong Kong, G/F Multicentre, Tai Po Hospital, 9 Chuen On Road, Tai Po, NT, Hong Kong; 3 Department of Psychology, Room 328 Sino Building, Chung Chi College, Chinese University of Hong Kong, Shatin, NT, Hong Kong; 4 Department of Applied Social Studies, City University of Hong Kong, 83 Tat Chee Avenue, Kowloon, Hong Kong; 5 Department of Medicine and Therapeutics, Chinese University of Hong Kong, 9/F Clinical Sciences Building, Prince of Wales Hospital, 30-32 Ngan Shing Street, Shatin, NT, Hong Kong; 6 Nethersole School of Nursing, 7/F Esther Lee Building, Chinese University of Hong Kong, Shatin, NT, Hong Kong

**Keywords:** Alzheimer’s disease, Burden, Depression, Positive aspects of caregiving, Positive reappraisal

## Abstract

**Background:**

Caregivers of relatives with Alzheimer’s disease are highly stressed and at risk for physical and psychiatric conditions. Interventions are usually focused on providing caregivers with knowledge of dementia, skills, and/or support, to help them cope with the stress. This model, though true to a certain extent, ignores how caregiver stress is construed in the first place. Besides burden, caregivers also report rewards, uplifts, and gains, such as a sense of purpose and personal growth. Finding benefits through positive reappraisal may offset the effect of caregiving on caregiver outcomes.

**Design:**

Two randomized controlled trials are planned. They are essentially the same except that Trial 1 is a cluster trial (that is, randomization based on groups of participants) whereas in Trial 2, randomization is based on individuals. Participants are randomized into three groups - benefit finding, psychoeducation, and simplified psychoeducation. Participants in each group receive a total of approximately 12 hours of training either in group or individually at home. Booster sessions are provided at around 14 months after the initial treatment. The primary outcomes are caregiver stress (subjective burden, role overload, and cortisol), perceived benefits, subjective health, psychological well-being, and depression. The secondary outcomes are caregiver coping, and behavioral problems and functional impairment of the care-recipient. Outcome measures are obtained at baseline, post-treatment (2 months), and 6, 12, 18 and 30 months.

**Discussion:**

The emphasis on benefits, rather than losses and difficulties, provides a new dimension to the way interventions for caregivers can be conceptualized and delivered. By focusing on the positive, caregivers may be empowered to sustain caregiving efforts in the long term despite the day-to-day challenges. The two parallel trials will provide an assessment of whether the effectiveness of the intervention depends on the mode of delivery.

**Trial registration:**

Chinese Clinical Trial Registry (http://www.chictr.org/en/) identifier number ChiCTR-TRC-10000881.

## Background

Alzheimer disease (AD) accounts for about two-thirds of all the dementia cases [1], and is a chronic, degenerative disease. The burden of caregiving often results in physical exhaustion, depression, and poor health [2-5], thus interfering with the ability of the caregiver (CG) to deliver care effectively. Recent evidence suggests that the stress of dementia caregiving increases morbidity in CGs via its immunosuppressive effect that is mediated by elevated levels of cortisol (a biochemical marker of stress) most noticeable in the morning, the afternoon and the evening [6-8]. In Hong Kong, over half of the caregivers are children; many CGs, in particular daughters and daughters-in-law, have to juggle between the responsibilities for the ill parent, children, work and other family matters. Noncognitive symptoms, that is, behavioral and psychological symptoms of dementia (BPSD) are consistently shown to be more stressful for caregivers than cognitive symptoms (for example, memory impairment) or functional impairment [9,10].

Interventions for dementia CGs usually include some form of psychoeducation, counseling, personal care training, social support, stress management, and home environment modification [11-15]. The objective of interventions is usually to strengthen CGs’ resources to deal with the challenges of caring for an AD family member on a day-to-day basis. These interventions have relied primarily on a deficiency model; that is, the CGs’ difficulties are due to their lack of knowledge, skills, and/or support. This model, though true to a certain extent, ignores how CG stress is construed in the first place.

### Rationale

Alongside the stressful day-to-day tasks, caregivers also report rewards, uplifts, and gains. CGs often report enhanced relationships with the care-recipient (CR) and others around them; insights about hardship, personal strength, and priorities in life; appreciation of life and its frailty; and a sense of purpose, sacrifice and devotion to doing what needs to be done. In the context of intergenerational caregiving, child CGs also report a sense of filial piety, awareness of aging, and more empathy for older adults [16-20]. In the stress-and-coping framework, such positive aspects of caregiving may be framed as positive reappraisal and meaning-focused coping [21], forms of emotion-focused coping that are especially suitable for uncontrollable events [22], such as the many stressors dementia caregivers face. With positive reappraisal, caregivers are expected to reduce appraisal of caregiving tasks as stressful and to develop a more balanced perspective of the caregiving experience [21]. Finding benefits through positive reappraisal may even offset the effect of BPSD on burden [23].

A key mechanism by which interventions lead to benefit finding is cognitive processing, which is ‘the process of actively thinking about a stressor, the thoughts and feelings it evokes, and its implications for one’s life and future’ [24]. Cognitive processing is a necessary but not sufficient condition for finding benefits. because not all caregivers who engage in cognitive processing find benefits or meanings. In fact, cognitive processing without finding benefits has no positive health effects [24], and may even lead to depressive mood if it is focused on negative aspects of the experience [25]. Hence it is important that cognitive processing is guided by positive reappraisal coping [21]. Research on cancer patients showed that finding benefits completely mediated the relationship between intervention and serum cortisol [26], and were associated with subsequent lymphocyte proliferation in cancer patients [27].

**Figure 1:**
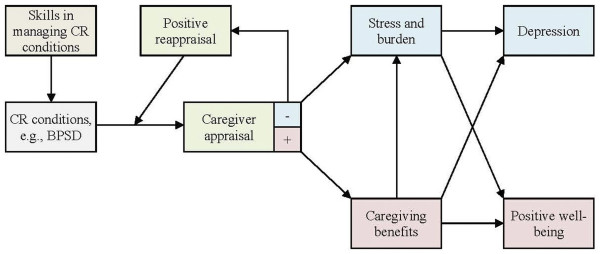
Theoretical model.

Our theoretical model is presented in Figure 1. In brief, caregiver outcomes are not the direct results of symptoms and dependency needs of the patient, but are mediated by appraisal. Whereas a positive appraisal would lead to positive well-being and growth, a negative appraisal leads to negative outcomes such as depression. The objective of our intervention is to increase the odds of positive appraisal over that of negative appraisal. This will be achieved by trainings on positive reappraisal. This intervention differs from traditional psychoeducational intervention that focuses on providing caregivers with the knowledge and skills to manage patient conditions such as BPSD and physical impairment. Besides psychoeducation, our benefit-finding intervention requires caregivers to practice positive reappraisals.

### Study objectives

The purpose of this randomized controlled trial is to evaluate the effectiveness of a new, multicomponent intervention combining psychoeducation with benefit finding. Two control groups will be used. First, in order to ascertain whether the benefit-finding component is useful, this intervention will be evaluated against a ‘pure’ psychoeducation program. However, the psychoeducation group used in this study is strictly speaking not a treatment-as-usual control because it is far more in-depth and comprehensive than the psychoeducation programs offered by service agencies in developing countries in this region, which typically are brief (for example, consisting of a few sessions) and focused on information provision (that is, education) only. From both scientific and policy points of view, a second control group that more closely mimics the existing services is needed. Thus, a simplified psychoeducation program focused only on information provision is created for this purpose. This allows the benefit-finding intervention to be evaluated against the form of psychoeducation that is commonly reported in the mainstream, Western literature as well as against the version of psychoeducation that is commonly found in this region. Trial 1 will evaluate the interventions in group settings (for example, social centers, clinics), whereas in Trial 2, the interventions will be conducted individually in the caregivers’ own homes. These two trials together permit an assessment of the effects of the interventions in different modes of delivery.

## Study design

### Participants

Primary CGs of a family member (aged 65+) with a physician diagnosis of AD or meeting the NINCDS-ADRDA criteria for possible AD [28] will be recruited from psychiatric/psychogeriatric clinics, NGOs, and the community. Primary CGs are those who provides the greatest amount of unpaid care (at no less than 14 hours per week) to the CR and is primarily responsible for making day-to-day care decisions. Care includes providing assistance in any of the following: feeding, dressing, appearance, moving around, bed transfer, bathing, toileting, using the telephone, transportation, shopping, preparing meals, housework, taking medication, and handling money. Other than CR diagnosis and the number of caregiving hours per week, the inclusion criteria are: (a) CR being in the mild to moderate range of dementia severity as determined by Clinical Dementia Rating [29], (b) CG aged 18+ years, and (c) CG without cognitive impairment (that is, Mini Mental State Examination (MMSE) ≥23) [30]. Exclusion criteria were CR having parkinsonism or other forms of dementia (vascular dementia, dementia with Lewy bodies, mixed dementia, and so on). Some 120 participants will be recruited for Trial 1 and 100 for Trial 2. Informed consent will be obtained from the caregivers. Ethics approval for the study was obtained from the Joint CUHK-NTEC Clinical Research Ethics Committee.

### Randomization and masking

A computer software generating true random numbers will be used for assigning participants into interventions. For Trial 1, participants will be randomized by center or clinic (that is, participants recruited from the same setting will be randomized in one unit). For Trial 2, participants will be randomized individually. In both trials, participants will be told on the consent form that they would receive ‘a course related to the caring of patients with dementia,’ without being told the different interventions. The trainers will not be used as interviewers so as to ensure that the interviewers are blind to the participants’ assignment status as well. Hence, both trials are double-blind randomized controlled trials, but Trial 1 differs from Trial 2 in being a cluster trial.

### Interventions

Trainers should have a background in psychology, social work, occupational therapy or related fields, with experiences in gerontology and dementia. Each treatment condition will last for eight weeks. In the group mode, this will be accomplished by eight weekly group interventions lasting one-and-a-half to two hours each. To facilitate interaction during group sessions, each group will have a maximum of 10 caregivers. In the individual mode, there will be four biweekly sessions of three hours each conducted at the caregiver’s home. The number of face-to-face contact hours between trainer and caregiver is held to be constant across the three intervention groups to control for possible dosage effects.

The psychoeducation group will receive information on aging in general, AD, BPSD, general coping skills (including relaxation, but not positive reappraisal), community resources, ways to handle BPSD and activities of daily living (ADL) needs, and communication skills with the CR. Application of skills will be discussed in relation to CRs’ needs and perspectives, and the importance of developing alternative approaches to match CR conditions.

The benefit-finding group will receive the same curriculum as psychoeducation (but not spending as much time on each topic) plus training in positive reappraisal coping and finding benefits. In addition, during this period, they will record ‘benefit diaries’ up to three times per week; during the evening, caregivers will reflect on the events and experiences in the past few days and mention three events/experiences from which benefits are constructed. Because literacy rates are low in the older population in Hong Kong, in order to standardize the diary method, all participants will be provided with a voice recorder to record their reflections. This also serves as a quality check that the diaries are produced by the participant personally.

Finally, the simplified psychoeducation group will receive an identical curriculum as the psychoeducational group, except that the application and practical components will be delivered in talks only, without hands-on exercises and follow-up discussions. In other words, lecture will be the primary mode of delivery, as is typical of local psychoeducation programs.

On the whole, the simplified psychoeducation group is characterized by information provision only. By comparison, the psychoeducation group is also focused on information provision but it is done in a way that facilitates daily application through dedicated discussions, practical exercises, and feasibility assessments that incorporate the potential needs and perspectives of the CR. Compared with psychoeducation, the benefit-finding group further adds various activities, including diaries, to promote positive reappraisal and finding positive gains in caregiving.

Further to the baseline interventions, boosters aimed to refresh and extend learning will be provided for all three groups at roughly 14 months after the conclusion of the initial treatment. The specific contents of the training programs are shown in Tables 1, 2, 3 and 4.

**Table 1:** Baseline intervention - Trial 1 (Group)^*^

	**Benefit-finding**	**Psychoeducation**	**Simplified psychoeducation**
Session 1 (week 1)	1. Introduce basic knowledge of dementia	1. Introduce basic knowledge of dementia	1. Introduce basic knowledge of dementia
	2. Discuss communication skills with CR, with illustrations		
	3. Introduce the diary task of benefit finding		
Session 2 (week 2)	1. Introduce concepts and skills in stress management	1. Introduce concepts and skills in stress management	1. Introduce concepts and skills in stress management
	2. Use ‘emotional thermometer’ to help them understand their emotional reactions in different situations	2. Balance between self-care and caregiving responsibilities	2. Balance between self-care and caregiving responsibilities
	3. Balance between self-care and caregiving responsibilities	3. Introduce importance of mutual support	3. Introduce importance of mutual support
	4. Introduce importance of mutual support	4. Relaxation exercise	4. Introduce relaxation without practice
	5. Introduce effects of cognition on emotion and behavior		
	6. Relaxation exercise		
Session 3 (week 3)	1. Introduce BPSD and possible causes	1. Introduce BPSD and possible causes	1. Introduce BPSD and possible causes
	2. Introduce ways to cope with and manage BPSD	2. Introduce ways to cope with and manage BPSD	2. Introduce ways to cope with and manage BPSD
	3. Introduce home-based activities for CR (for example, cognitive stimulation, multisensory stimulation, reminiscence)	3. Introduce home-based activities for CR (for example, cognitive stimulation, multisensory stimulation, reminiscence)	3. Introduce home-based activities for CR (for example, cognitive stimulation, multisensory stimulation, reminiscence)
	4. Introduce and practice positive reappraisal; mutual sharing of benefits	4. Use case study to illustrate behavioral management	
	5. Use case study to illustrate behavioral management and emotion-focused coping, with particular emphasis on positive reappraisals		
Session 4 (week 4)	1. Repeat Session 3 on behavioral management and positive reappraisal with different BPSD; additional examples and case study are presented	1. Repeat Session 3 on psychological symptoms management; additional examples and case study are presented	1. Repeat Session 3 on psychological symptoms management; additional examples are presented
Session 5 (week 5)	1. Introduce caring skills for ADL I - bathing, dressing, and incontinence, and so on	1. Introduce caring skills for ADL I - bathing, dressing, and incontinence, and so on	1. Introduce caring skills for ADL I - bathing, dressing, and incontinence, and so on
	2. Transfer and support techniques with practice	2. Transfer and support techniques with practice	2. Demonstrate transfer and support techniques without practice
	3. Sharing by child caregiver on caregiving experience and benefits to self		
Session 6 (week 6)	1. Introduce caring skills for ADL II - taking medications, use of telephone, housework, and so on	1. Introduce caring skills for ADL II - taking medications, use of telephone, housework, and so on	1. Introduce caring skills for ADL II - taking medications, use of telephone, housework, and so on
	2. Sharing by spouse caregiver on caregiving experience and benefits to self	2. Introduce timetabling and activity planning	2. Introduce timetabling and activity planning
	3. Introduce timetabling and activity planning		
Session 7 (week 7)	1. Home environment	1. Discuss communication skills with CR, with illustration	1. Discuss communication skills with CR, with illustration
	2. Community resources		
	3. Practice positive reappraisal; mutual sharing of benefits		
Session 8 (week 8)	1. Sharing by child-in-law caregiver on caregiving experience and benefits to self	1. Home environment	1. Home environment
	2. Acceptance of caregiving role	2. Community resources	2. Community resources
	3. Reflect on motivations to provide care		
	4. Overview of things learned		
	5. Set goals for improvement in coming year, with particular reference to areas where perceived benefits are still lacking		

*The full training manuals include detailed instructions, PowerPoint presentations, and videos.

**Table 2:** Booster intervention - Trial 1 (Group)^*^

	**Benefit-finding**	**Psychoeducation**	**Simplified psychoeducation**
Session 1 (week 1)	1. Review home-based activities for CR with illustration	1. Review home-based activities for CR with illustration	1. Review home-based activities for CR
	2. Revise and practice positive reappraisal; mutual sharing of benefits	2. Relaxation exercise	2. Revise relaxation instructions without practice
	3. Review progress in meeting goals set one year earlier		
	4. Relaxation exercise		
Session 2 (week 2)	1. Revise knowledge of severe AD, including end stage	1. Revise knowledge of severe AD, including end stage	1. Revise knowledge of severe AD, including end stage
	2. Anticipatory grief	2. Anticipatory grief	2. Anticipatory grief
	3. Advanced medical directive	3. Advanced medical directive	3. Advanced medical directive
	4. Community resources, for example, palliative care	4. Community resources, for example, palliative care	4. Community resources, for example, palliative care
	5. Set goals to achieve prior to CR death	5. Relaxation exercise	
	6. Relaxation exercise		
Session 3 (week 3)	1. Review other knowledge of dementia, stress management, ADL care skills, and BPSD management	1. Review other knowledge of dementia, stress management, ADL care skills, and BPSD management	1. Review other knowledge of dementia, stress management, ADL care skills, and BPSD management
	2. Revisit the importance of timetabling and activity planning with exercise and discussions to reinforce application	2. Revisit the importance of timetabling and activity planning with exercise and discussions to reinforce application	2. Revisit the importance of timetabling and activity planning without exercise and discussion
	3. Practice positive reappraisal; mutual sharing of benefits	3. Relaxation exercise	
	4. Relaxation exercise		
Session 4 (week 4)	1. More on community resources	1. More on community resources	1. More on community resources
	2. Remember activities with CR and relationship with him/her - construct positive meanings through photo narrative	2. Remember activities with CR and relationship with him/her through photo narrative	2. Introduce photo narrative
	3. Practice positive reappraisal; mutual sharing of benefits	3. Relaxation exercise	
	4. Relaxation exercise		

*The full training manuals include detailed instructions, PowerPoint presentations, and videos.

**Table 3:** Baseline intervention - Trial 2 (Individual)^*^

	**Benefit-finding**	**Psychoeducation**	**Simplified psychoeducation**
Session 1 (week 1)	1. Introduce basic knowledge of dementia	1. Introduce basic knowledge of dementia	1. Introduce basic knowledge of dementia
	2. Introduce BPSD and possible causes	2. Introduce BPSD and possible causes	2. Introduce BPSD and possible causes
	3. Introduce ways to cope with and manage BPSD	3. Introduce ways to cope with and manage BPSD	3. Introduce ways to cope with and manage BPSD
	4. Use ‘emotional thermometer’ to help them understand their emotional reactions in different situations	4. Introduce home-based activities for CR (for example, cognitive stimulation, multisensory stimulation, reminiscence)	4. Introduce home-based activities for CR (for example, cognitive stimulation, multisensory stimulation, reminiscence)
	5. Introduce effects of cognition on emotion and behavior	5. Use case study to illustrate behavioral management	
	6. Introduce the diary task of benefit finding		
Session 2 (week 3)	1. Repeat Session 1 on behavioral management and positive reappraisal with different BPSD; additional examples and case study are presented	1. Introduce caring skills for ADL I - bathing, dressing, and incontinence, and so on	1. Introduce caring skills for ADL I - bathing, dressing, and incontinence, and so on
	2. Introduce home-based activities for CR (for example, cognitive stimulation, multisensory stimulation, reminiscence)	2. Transfer and support techniques with practice	2. Demonstrate transfer and support techniques without practice
	3. Use case study to illustrate behavioral management and emotion-focused coping, with particular emphasis on positive reappraisals	3. Introduce caring skills for ADL II - taking medications, use of telephone, housework, and so on	3. Introduce caring skills for ADL II - taking medications, use of telephone, housework, and so on
	4. Introduce and practice positive reappraisal; mutual sharing of benefits	4. Additional examples and case study are presented	
	5. Reflect on motivations to provide care		
	6. Discuss communication skills with CR, with illustrations		
	7. Sharing by child caregiver on caregiving experience and benefits to self		
	8. Home environment		
Session 3 (week 5)	1. Introduce caring skills for ADL I - bathing, dressing, and incontinence, and so on	1. Discuss communication skills with CR, with illustration	1. Discuss communication skills with CR, with illustration
	2. Transfer and support techniques with practice	2. Home environment	2. Home environment
	3. Practice positive reappraisal; mutual sharing of benefits	3. Introduce timetabling and activity planning	3. Introduce timetabling and activity planning
	4. Sharing by child-in-law caregiver on caregiving experience and benefits to self		
	5. Introduce caring skills for ADL II - taking medications, use of telephone, housework, and so on		
	6. Introduce timetabling and activity planning		
	7. Community resources		
Session 4 (week 7)	1. Introduce concepts and skills in stress management	1. Introduce concepts and skills in stress management	1. Introduce concepts and skills in stress management
	2. Acceptance of caregiving role	2. Balance between self-care and caregiving responsibilities	2. Balance between self-care and caregiving responsibilities
	3. Balance between self-care and caregiving responsibilities	3. Introduce importance of mutual support	3. Introduce importance of mutual support
	4. Introduce importance of mutual support	4. Relaxation exercise	4. Introduce relaxation without practice
	5. Overview of things learned	5. Community resources	5. Community resources
	6. Relaxation exercise		
	7. Sharing by spouse caregiver on caregiving experience and benefits to self		
	8. Set goals for improvement in coming year, with particular reference to areas where perceived benefits are still lacking		

*The full training manuals include detailed instructions, PowerPoint presentations, and videos.

**Table 4:** Booster intervention - Trial 2 (Individual)^*^

	**Benefit-finding**	**Psychoeducation**	**Simplified psychoeducation**
Session 1 (week 1)	1. Review home-based activities for CR with illustration	1. Review home-based activities for CR with illustration	1. Review home-based activities for CR
	2. Revise and practice positive reappraisal; review selected examples of benefit provided by other caregivers	2. Review other knowledge of dementia, stress management, ADL care skills, and BPSD management	2. Review other knowledge of dementia, stress management, ADL care skills, and BPSD management
	3. Review progress in meeting goals set one year earlier	3. Revisit the importance of timetabling and activity planning with exercise and discussions to reinforce application	3. Revisit the importance of timetabling and activity planning without exercise and discussion
	4. Review other knowledge of dementia, stress management, ADL care skills, and BPSD management	4. Relaxation exercise	4. Revise relaxation instructions without practice
	5. Revisit the importance of timetabling and activity planning with exercise and discussions to reinforce application		
	6. Practice positive reappraisal; review selected examples of benefit provided by other caregivers		
	7. Relaxation exercise		
Session 2 (week 3)	1. Revise knowledge of severe AD, including end stage	1. Revise knowledge of severe AD, including end stage	1. Revise knowledge of severe AD, including end stage
	2. Anticipatory grief	2. Anticipatory grief	2. Anticipatory grief
	3. Advanced medical directive	3. Advanced medical directive	3. Advanced medical directive
	4. Community resources, for example, palliative care	4. Community resources, for example, palliative care	4. Community resources, for example, palliative care
	5. Set goals to achieve prior to CR death	5. More on community resources	5. More on community resources
	6. More on community resources	6. Remember activities with CR and relationship with him/her through photo narrative	6. Introduce photo narrative
	7. Remember activities with CR and relationship with him/her - construct positive meanings through photo narrative	7. Relaxation exercise	
	8. Practice positive reappraisal; review selected examples of benefit provided by other caregivers		
	9. Relaxation exercise		

*The full training manuals include detailed instructions, PowerPoint presentations, and videos.

An important consideration when running interventions at centers or clinics is that the CRs will need to be looked after simultaneously when the caregivers attend the training. Thus, volunteer or student assistants will be needed to look after and run activities for the CRs with AD. We recommend a ratio of three assistants to one CR.

### Adverse events

It is anticipated that no adverse effects due to the training will occur. This is consistent with trials on caregiver training around the world.

### Outcome measures

The primary outcomes are CG stress (subjective burden [31,32], role overload [33], and cortisol), perceived benefits (from open-ended questions), subjective health [34], psychological well-being [35,36], and depression [37,38]. The secondary outcomes are CG coping [39], and CR’s BPSD [40,41] and functional impairment [42,43]. Same for group and individual interventions, all outcome measures, except cortisol, will be obtained at baseline (0 month; T1), post-treatment (2 months; T2), and 6 (T3), 12 (T4), 18 (T5) and 30 months (T6). Salivary cortisol, collected using the Salivette five times during the day (immediately after awakening, 30 minutes post-awakening, 45 minutes post-awakening, and at 11:00 and 21:00) for two consecutive days, will be obtained at T1-T4 only. This design will allow an assessment of the long-term effects of the interventions. The last assessment (T6) is not only necessary to assess the more long-term impacts of the treatment, but is spaced out at a longer interval in order to provide a more stringent test of the effects of the treatment. It is possible that the half-yearly assessment conducted from six months onward may serve as soft reminders for benefit searching, from which the benefit group may gain more. The 12-month interval from T5 to T6 eliminates this possibility and enhances the generalizability of the findings to naturalistic settings. Data analysis will be performed using the principle of intent-to-treat, and missing values will be estimated by multiple imputations using Markov chain Monte Carlo method.

### Study hypotheses

We expect that participants in the benefit-finding group will report more benefits, lower burden, lower overload, better subjective health, better psychological well-being, and lower depression than both the psychoeducation and the simplified psychoeducation group, whereas the latter two would not differ from each other. For cortisol, the benefit-finding group is expected to have a steeper slope of diurnal decline and lower cortisol level at 21:00 than the other two groups. With respect to cortisol in the awakening period, the benefit-finding group is expected to have higher levels at 0, +30 and +45 minutes post-awakening than the other two groups. We also predict an improvement in CG coping, CR BPSD, and a slower rate of decline of CR functional impairment for the benefit-finding group, over the other two groups, over time.

### Statistical analysis

The data structures contain multiple levels. For Trial 1, repeated measurements (level 1) is nested within persons (level 2), which then is nested with the cluster (level 3) from which they were recruited. For Trial 2, the data contain the first two levels only. Because of the multilevel structure, data will be analyzed using mixed-effects regression with full-information maximum-likelihood estimation in Stata version 11.1. This procedure does not require imputation of missing data common in longitudinal studies because the conditional distribution of missing data based on data observed are incorporated into estimation of parameters [44].

In mixed-effects regression, the intercepts and effect of time (centered) will be specified to vary randomly at cluster (Trial 1) and person (both Trial 1 and Trial 2) levels. Two dummies will be created for the treatment groups with simplified psychoeducation as the reference category. Thus the effects of benefit-finding and psychoeducation will be compared against simplified psychoeducation in the first set of analyses. Product terms of benefit-finding x time and psychoeducation x time will be created to test whether changes in the outcome measures over time are different from those of the simplified psychoeducation group. Additional analyses will be conducted by dropping participants in the simplified psychoeducation condition while coding psychoeducation as the reference group, so that the effects of benefit-finding can be compared directly with psychoeducation as well. In this case, only two treatment groups will be included in the analyses.

With six repeated measurements (month 0, 2, 6, 12, 18, and 30), a medium treatment by time interaction effect (Cohen’s *d* = 0.50), power = 0.80, two-tailed alpha = .017 (Bonferroni-corrected for multiple comparison among three treatment groups), ratio of time-effect variance at person-level to the sum of random-intercept variance and time-level residual variance = 0.20 (which is quite large to yield conservative estimates of power), 17 participants per experimental condition are sufficient, no matter whether there are two or three experimental conditions in the regression analysis. For the cluster-randomized controlled trial, with the addition of the ratio of time-effect variance at home-level to the sum of random-intercept variance and time-level residual variance = 0.02, four homes per condition and 10 participants per home are adequate, regardless of whether there are two or three experimental conditions in the regression analysis [45,46].

## Discussion

Given the global rise in dementia incidence in the coming decades [47], ways to support informal caregivers will no doubt become a major health-care agenda item around the world. Without the unpaid work of family CGs, the burden on social and health services will be tremendous. Although some caregivers may benefit from services like day care [48,49], the fact remains that the majority of caregivers do not seek help [50] and need to face the round-the-clock demands of caregiving mostly on their own. Researchers need to continue experimenting with new and more effective ways to support caregivers. In our approach under investigation, the emphasis on benefits rather than losses and difficulties provides a new dimension to the way interventions for caregivers can be conceptualized and delivered. This approach is consistent with the life-span developmental perspective [51], which suggests that successful adaptations do not require the absence of losses, but a more positive balance of gains over losses. Our model is the first attempt to apply such a theoretical perspective to the clinical studies of caregiver burden. By focusing on the positive, caregivers may be empowered to sustain caregiving efforts in the long term despite the day-to-day challenges, with less adverse outcomes or even positive outcomes in the long term. The two parallel trials will also provide an assessment of whether the effectiveness of the intervention depends on the mode of delivery.

## Trial status

Trial 1 started in August 2010 and Trial 2 started in February 2011. By 26 January 2012, we had recruited 112 and 86 participants for Trial 1 and Trial 2, respectively. These participants had also completed the respective intervention to which they were assigned, and were being followed up. By this time, six and two individuals had dropped out of Trial 1 and Trial 2, respectively. Both trials are expected to be completed around May 2014.

## Abbreviations

AD, Alzheimer disease; ADL, Activities of daily living; BPSD, Behavioral and psychological symptoms of dementia; CG, Caregiver; CR, Care-recipient.

## Competing interests

The authors declare that they have no competing interests.

## Authors’ contributions

STC, LCWL, JCLL, TK, HHF, and DTFL contributed to initial conceptualization and design, and were part of the research team that secured funding, with STC being the principal investigator who oversaw and managed the entire project. STC, RWLL, EPMM, and NSSN, and LCWL contributed to the training manual. LCWL, RWLL, EPMM, NSSN, and TK recruited participants, while RWLL, EPMM, and NSSN collected the data. All contributed to the writing, with STC taking the primary responsibility. All authors read and approved the final manuscript.
